# Privacy versus safety in contact-tracing apps for coronavirus disease 2019

**DOI:** 10.1177/2055207620941673

**Published:** 2020-07-14

**Authors:** Pierfrancesco Lapolla, Regent Lee

**Affiliations:** Nuffield Department of Surgical Sciences, University of Oxford, UK

With a view to a gradual exit from lockdown, governments around the world are considering deploying contact-tracing apps to prevent or manage a second wave of coronavirus disease 2019 (COVID-19).

Through smartphones, contact-tracing apps can identify people who may have come in contact with an infected person. Based on Bluetooth Low Energy (BLE) and with optional geo-localisation (GPS), this technology can track people’s movements. When an infected subject is close enough to another person, the latter becomes a potential infected case who can be contacted and tracked. The aim is to isolate the potentially infected cases to reduce the spread of COVID-19.^1^

Many concerns arise over efficacy, privacy issues and data management by governments or health authorities. Another crucial concern is the cybersecurity of the app-supporting infrastructures which can be exposed to third-party attacks. A simulation on one million people found that 80% of smartphone users in the UK (56% of the general population) would need to install a contact-tracing app to suppress the epidemic effectively.^2^ A survey run in five countries with more than 6000 potential app users suggested that lower numbers would install a similar app (73.6% of users in the UK, and 67.5–85.5% in France, Germany, Italy and the USA).^2^ In Singapore, the first country to deploy a voluntary contact-tracing app (TraceTogether), launched in March, only an estimated 17% of the population installed the app.^3^ After a spike in new cases in April, the city-state introduced a lockdown named ‘circuit breaker’. Regarding the digital alphabetisation aspect, data for the Italian population in 2019 show that only 29.1% of the 16–74 age group of Internet users have high digital skills; the majority of Internet users have low (41.6%) or basic (25.8%) skills. Not only that, but it is more the younger population in Italy who have smartphones rather than mobiles.^4^

Currently, various different frameworks have been developed to build contact tracing, such as open frameworks (GA-PPTP, DP-3T, Blue Trace, TCN) or private and controlled (PEPP-PT). The nature of implementation may be open source (DP-3T, Blue Trace, TCN) or private (PEPP-PT, GA-PPTP), and the control-based network can be decentralised or centralised proximity data. On 19 April, a letter signed by nearly 300 academics warned that centralised systems can risk surveillance, and suggested that Apple and Google (currently working jointly in developing a contact-tracing app) should consider developing one which uses an opt-in and decentralised system.^5^

Countries such as China,^6^ Singapore and Colombia have officially adopted contact-tracing apps.^7,8^ Controversies arise over app security issues and data breach (as was the case in The Netherlands),^9^ especially for apps including geo-localisation (such as the ones deployed in Norway and Israel).^10,11^

Contact-tracing apps might be an effective way of controlling the pandemic through the next phases. However, in order to be effective, contact tracing must be supported not only by solid technology, capable of minimising the risk of attacks, but also by a system offering safe communication with appropriate authorities. Therefore, concerted pan-European efforts to resolve concerns over the privacy implications will be essential in the development of successful COVID-19 contact-tracing apps.
